# Portal Vein Thrombosis

**DOI:** 10.1155/2015/823063

**Published:** 2015-02-23

**Authors:** Ronny Cohen, Thierry Mallet, Michael Gale, Remigiusz Soltys, Pablo Loarte

**Affiliations:** ^1^Woodhull Medical Center, 760 Broadway, Brooklyn, NY 11206, USA; ^2^NYU Langone Medical Center and School of Medicine, 550 First Avenue, New York, NY 10016, USA; ^3^Department of Medicine, Woodhull Medical Center, 760 Broadway, Brooklyn, NY 11206, USA; ^4^St. Georges University, School of Medicine, West Indies, Grenada; ^5^Department of Internal Medicine, Yale-New Haven Hospital, 20 York Street, New Haven, CT 06510, USA

## Abstract

Portal vein thrombosis (PVT) is the blockage or narrowing of the portal vein by a thrombus. It is relatively rare and has been linked with the presence of an underlying liver disease or prothrombotic disorders. We present a case of a young male who presented with vague abdominal symptoms for approximately one week. Imaging revealed the presence of multiple nonocclusive thrombi involving the right portal vein, the splenic vein, and the left renal vein, as well as complete occlusion of the left portal vein and the superior mesenteric vein. We discuss pathogenesis, clinical presentation, and management of both acute and chronic thrombosis. The presence of PVT should be considered as a clue for prothrombotic disorders, liver disease, and other local and general factors that must be carefully investigated. It is hoped that this case report will help increase awareness of the complexity associated with portal vein thrombosis among the medical community.

## 1. Introduction

Portal vein thrombosis (PVT) is the blockage or narrowing of the portal vein by a thrombus. It is relatively rare and has been linked with the presence of an underlying liver disease or prothrombotic disorders. However, no cause is identified in more than 25% of patients [1]. Since a lot of the patients are asymptomatic, the diagnosis is usually made in the presence of complications. Acute and chronic versions of this entity are considered, although a clear clinical distinction may be difficult [2]. Abdominal pain is an interesting presentation of portal vein thrombosis, such as in the patient that will be presented in the following case report. Regarding the management, despite the lack of large randomized trials, a consensus on optimal treatment is being sought, since recent studies seem to indicate the efficacy of thrombolysis in acute cases and the benefit of anticoagulation in patients with chronic portal vein thrombosis [2].

## 2. Case Presentation

A 25-year-old man presented to the ER with abdominal pain, nausea, and vomiting. He described the abdominal pain as cramping, constant, located on the left lower side of the abdomen with an intensity of 8 to 9 out of 10. He indicated that the pain had been present for 1 week and had been getting progressively worse, with no radiation to other areas of the abdomen. Associated symptoms reported were nausea and vomiting for 1 day, diarrhea that had been on and off, and fatigue for about a week. He denied any fever, chest pain, shortness of breath, constipation, dizziness, muscle weakness, or numbness.

His past medical history was significant for bipolar disorder and dyslipidemia. No surgical history was reported. The patient denied any allergies. He was taking gabapentin and mirtazapine. His family history is unknown, since he was adopted at the age of 5. He reported occasional alcohol and tobacco use and had used cocaine and marijuana in the past, with the last use reported to be 1 year prior to admission, since he was currently enrolled in a methadone program. He was unemployed at the time of admission. Interestingly, he reported having been admitted to a hospital 2 years prior where he was told he had some blood clots in his abdomen and that he needed to take blood thinners, an advice that he did not follow.

On presentation, the patient was in mild distress because of abdominal pain; the vital signs were stable, except for slight tachycardia with a heart rate of 106. No abnormal coloration was noted in the eyes or on the skin. No murmurs or additional heart sounds were noted. The lungs were clear to auscultation. The abdomen was soft and nondistended but was diffusely tender to palpation, especially in the left lower quadrant. No edema was noted in the extremities and the pulses were present. Neurological exam was intact.

**Figure 1:**
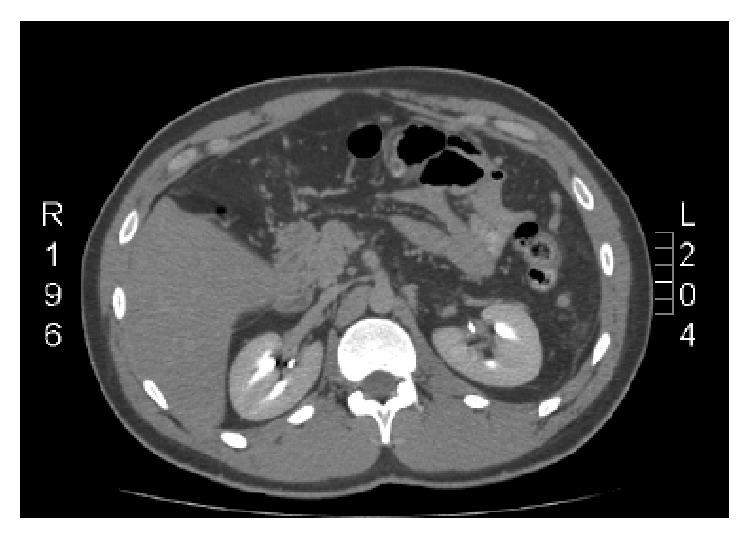
Left renal vein thrombosis with filling defect.

**Figure 2:**
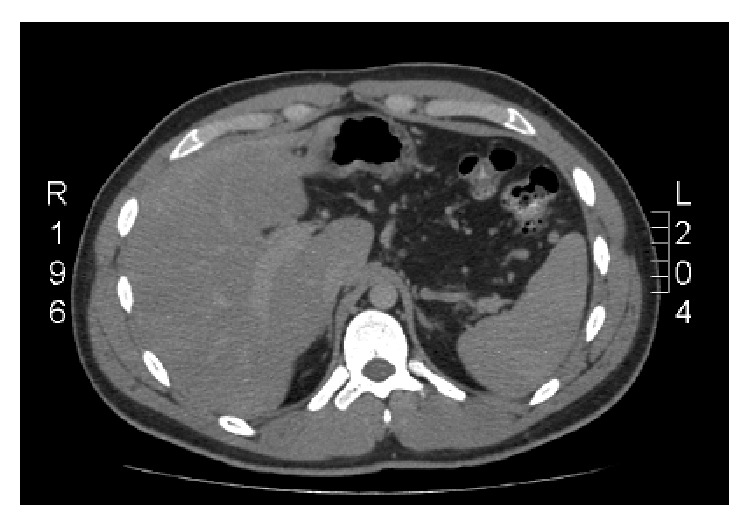
Left branch of the portal vein not seen, likely obstructed by a thrombus (only the right branch is appreciated).

The laboratory evaluation was basically unremarkable and revealed a WBC count of 8.89 (10^3^/*μ*L), Hb 15.2 g/dL, Hct 43.7%, Plt 248 (10^3^/*μ*L), INR within normal limits, Na 141 mEq/L, K 4.1 mEq/L, BUN 8 mg/dL, and Cr 1.2 mg/dL, Alk. Phos. 192 U/L. However, the radiological exam was significant, with an abdominal computed tomography (CT) revealing the presence of multiple nonocclusive thrombi involving the right portal vein, the splenic vein, and the left renal vein, as well as complete occlusion of the left portal vein and the superior mesenteric vein (Figures 1 and 2). Colitis involving the descending and sigmoid colon was also noted and was believed to be ischemic in nature. A Doppler ultrasound was obtained and confirmed the findings suggestive of chronic portal vein thrombosis.

The patient was admitted with a diagnosis of portal vein thrombosis and was started on anticoagulation with enoxaparin bridged with warfarin. A complete hypercoagulability workup was obtained, all of it coming back negative. Liver disease was also excluded, with all the liver disease workup being unremarkable as well. Patient's pain was relieved with morphine. His hospital course was uncomplicated, with significant improvement of his pain and the ability to tolerate a regular diet without any gastrointestinal symptoms, such as nausea, diarrhea, or constipation. He was extensively counseled on the need to be compliant with his anticoagulation regimen to prevent the recurrence of acute thrombosis and was uneventfully discharged with appointments to follow at the medicine and warfarin clinics. Interestingly, the patient was readmitted 2 weeks later after an INR check revealed it to be supratherapeutic at 12.8. No signs of bleeding were noted, he received a dose of Vitamin K, and his warfarin was held. After a couple of days of observation, he was discharged with instructions to follow at the warfarin clinic for readjustment of his anticoagulation regimen.

## 3. Discussion

Portal vein thrombosis refers to the development of thrombosis within the extra-hepatic portal venous system draining into the liver [2]. It has been classified into 4 anatomic groups:

1. thrombosis confined to the portal vein beyond the confluence of the splenic and superior mesenteric vein (SMV);
2. extension of thrombus into the SMV but with patent mesenteric vessels;
3. diffuse thrombosis of splanchnic venous system but with large collaterals;
4. extensive splanchnic venous thrombosis but with only fine collaterals [3].

Based on this classification, our patient had category 2 portal vein thrombosis.

## 4. Predisposing Factors

The most frequent underlying factors are liver disease, prothrombotic disorders, and miscellaneous factors [2]. The most common etiologic factors of portal vein thrombosis are as follows:

- cirrhosis/portal hypertension;
- prothrombotic tendency;
- malignancy (local/distant);
- sepsis (local/systemic);
- schistosomiasis;
- pancreatitis;
- postsurgical (e.g., liver transplantation and splenectomy);
- umbilical vein catheterization;
- portal vein compression by nodes (e.g., TB and lymphoma);
- drugs (e.g., oral contraceptive);
- pregnancy/postpartum.

Among the miscellaneous conditions, sepsis is worth mentioning, since a large retrospective study has identified abdominal sepsis as a risk factor in 11% of PVT cases [4].

## 5. Pathogenesis

According to a recent hypothesis, venous thrombosis in general occurs only when several factors are combined [5]. These factors comprise inherited or acquired prothrombotic disorders, other thrombophilic factors, and local factors [6]. This general multifactorial theory seems to apply well to portal vein thrombosis, with general thrombophilic factors identified in approximately 60% of patients with PVT and local factors in 40% [6, 7]. The most common prothrombotic factors associated with portal vein thrombosis (PVT) are as follows:

- myeloproliferative disorders (e.g., polycythemia rubra vera, essential thrombocytosis, and myelofibrosis);
- antiphospholipid syndrome;
- anticardiolipin antibody;
- proteins C and S and antithrombin III deficiency;
- Factor V Leiden deficiency;
- G20210A prothrombin gene mutation;
- hyperhomocysteinemia;
- Paroxysmal nocturnal hemoglobinuria.

The local factors favoring or precipitating development of portal vein thrombosis can be further divided into 3 categories: conditions characterized by local inflammation with or without a systemic inflammatory response, surgical injury to the portal venous system, and malignancy involving the abdominal organs resulting in tumorous invasion or constriction of the portal venous system [6] as follows:

- local inflammatory lesions:
  - neonatal omphalitis;
  - diverticulitis;
  - appendicitis;
  - pancreatitis;
  - duodenal ulcer;
  - cholecystitis;
  - tuberculous lymphadenitis;
- injury to the portal venous system:
  - surgical portocaval shunting;
  - splenectomy;
  - colectomy;
  - gastrectomy;
- cancer of abdominal organs.

It seems that a combination of general and local factors is needed to enable the development of PVT, thus establishing the importance of a thorough investigation of those factors when facing a diagnosis of PVT.

## 6. Clinical Features

Two broad clinical categories have been considered, acute and chronic PVT. However, in the practice, it may be very difficult to distinguish between the types [2]. A more useful distinction would be to differentiate those with recent onset PVT from those with chronic disease. No definitive time frame distinguishes acute from chronic PVT, but it is generally accepted that patients who developed symptoms less than 60 days prior to hospital assessment should be considered as having acute or recent onset disease [2, 8].

The typical presentation of acute PVT is abdominal pain, nausea, and fever. The severity of symptoms may correlate with the extent of mesenteric venous thrombosis because of associated bowel ischemia [2, 9], and up to 10% of cases of bowel ischemia are due to mesenteric venous thrombosis [2, 10]. The acute onset of ascites may also be seen. The absence of clinical or radiological evidence of portal hypertension generally suggests PVT of recent onset.

Chronic PVT most frequently presents with problems related to portal hypertension, including gastrointestinal bleeding, splenomegaly, and hypersplenism. Ascites rarely occurs in the absence of established liver disease [2]. In a study by Janssen et al. [11], the overall 10-year survival in adult patients with established PVT was 54%, with survival increasing to 81% in those without cirrhosis, cancer, or mesenteric vein thrombosis, indicating that concomitant disease was a more important cause of death than PVT itself, even in the patients who presented with variceal bleeding.

Several complications including esophageal and gastric varices, portal hypertensive gastropathy, bleeding, and ascites may develop [12]. Variceal bleeding remains the most common complication during the natural course of the disease and is the presenting problem in approximately 30% of patients with PVT not related to established liver disease [2]. Some abnormalities of extra-hepatic biliary tree may also develop, especially in those with chronic PVT. The main explanation is the development of an extensive collateral circulation called portal cavernoma around the biliary tree [13], which can cause biliary compression and structuring resulting in cholangitis, cholelithiasis, and cholecystitis [14].

## 7. Diagnosis

Diagnosis should be suspected clinically in many different situations: abdominal pain, abdominal sepsis, and gastrointestinal bleeding (due to portal hypertension or fortuitous finding of portal hypertension) [6]. A range of imaging modalities may be used in the diagnosis of PVT, and an accurate diagnosis can be made in most cases using Doppler ultrasound, contrast-enhanced computer tomography (CT), or magnetic resonance angiography (MRA) [15]. More invasive techniques like carbon dioxide portography or intra-arterial digital subtraction angiography are generally not needed. CT scanning may be more useful than Doppler ultrasound in demonstrating portosystemic collaterals and the development of a cavernoma, both suggestive of a well-established PVT [2, 6]. Noninvasive imaging is also less reliable at diagnosing thrombus extension into the mesenteric vasculature [2]. Endoscopic ultrasound has also recently been shown to be sensitive and specific in the diagnosis of PVT [16].

## 8. Management

After establishing the diagnosis, it is important to determine when the thrombosis has developed and whether or not precipitating factors could be identified [6]. This will help to clarify the goals for the treatment, which will essentially be to reverse or prevent the advancement of thrombosis and/or to treat the complications of established PVT [2]. Those complications will most specifically consist of gastrointestinal varices, portal hypertension, and biliary complications.

A crucial aspect in the management of PVT is the question of anticoagulation. The effectiveness of anticoagulation in patients with evidence of acute PVT has been reported in a number of small studies and case reports [17, 18]. Those studies suggest that anticoagulation may result in recanalization in more than 80% of cases. Thrombolytic therapy has also been reported to lead to resolution of acute PVT [19], but its effectiveness was mostly seen in the patients who received treatment within 14 days of initial symptoms.

The duration of anticoagulation has not been standardized, but it may be pragmatic to adopt the management algorithm, as applied to deep vein thrombosis in the lower limb [20]. When a reversible cause is identified, a treatment course of 3 to 6 months, with maintenance of the INR at 2-3, may be appropriate. When a prothrombotic tendency is identified, there may be advantage in continued anticoagulation. Extensive thrombosis, including involvement of the splanchnic bed, may also justify long-term treatment [2].

However, more controversy has surrounded the role of anticoagulation in patients with chronic PVT. It certainly reflects concern about the use of anticoagulation in the setting of gastroesophageal varices. Recent studies by Condat et al. [4] showed that there was no difference in the bleeding rate, hemoglobin level, or subsequent transfusion requirement between those patients taking anticoagulation and those not taking anticoagulation in a population with no cirrhosis or malignancy. Interestingly, the use of anticoagulant therapy was associated with a significant reduction in new thrombotic episodes. A pragmatic approach would be to endoscopically eradicate the varices prior to commencing anticoagulation [2].

Management of PVT requires the physician to also think about the possible complications of PVT. For prophylaxis of a variceal bleed, recent studies have suggested that variceal band ligation is as effective as B-blockade for the prevention of a first bleed [21]. Both modalities have also been shown to reduce the rate of rebleeding as secondary prophylaxis. However, in patients with PVT, complete endoscopic eradication of varices following an initial bleed has been shown to significantly reduce the risk of recurrent bleeding, with a 5-year survival of 95% and no mortality related to recurrent bleeding, making this procedure the best method for secondary prophylaxis [22].

For the management of biliary complications in patients with PVT, intervention will only be indicated in those with clinical manifestations of biliary obstruction [2]. Decompression of the hypertensive portal venous system, with either transjugular intrahepatic portosystemic shunt (TIPSS) or surgical portosystemic shunting, has been shown to reduce biliary structuring [23], but the main therapy will be again endoscopy. Surgical approaches have fallen out of favor with the emergence of endoscopic therapy, especially because of the high mortality in surgical patients compared with the low mortality with effective medical and endoscopic therapy [2].

## 9. Case Synopsis

The patient presented in this case report likely had an episode of acute PVT superimposed on chronic PVT contracted likely a couple of years before presentation. The acute symptoms were present for about 1 week. The radiological findings seemed to indicate the presence of chronic PVT, even if no sign of portal hypertension was mentioned. The history of abdominal thrombus in the past combined with the patient's noncompliance with anticoagulation seemed to have precipitated the formation of another thrombus. No cause could be elicited after investigating the possible causes (the hypercoagulability workup and liver disease workup came back negative). Ischemic colitis was likely the consequence of the extension of the thrombus to the SMV and should be confirmed via biopsy. Since it is likely the second episode of idiopathic PVT, continued anticoagulation might be indicated. Endoscopic surveillance for development of gastroesophageal varices will need to be established.

## 10. Conclusion

Portal vein thrombosis is a rare disease, but our understanding of this disorder has improved during the last few years. The presence of PVT should be considered as a clue for prothrombotic disorders, liver disease, and other local and general factors that must be carefully investigated. Early anticoagulation seems to restore the vascular permeability in the majority of the cases. The management of possible complications like varices, portal hypertension, and biliary complications via endoscopic surveillance is key. It is hoped that this case report will help increase awareness of the complexity associated with portal vein thrombosis among the medical community.
